# “They look you up and down like you are nothing”: A qualitative exploration of sex workers’ health needs and interactions with UK healthcare services

**DOI:** 10.1016/j.ijnsa.2025.100392

**Published:** 2025-07-30

**Authors:** E. Molloy, R. Tiwana, B.c French, C. Christie, H. Smailes, J. Taylor, C. Bradbury-Jones, Co-Researcher 1Co-Researcher 2

**Affiliations:** a Department of Applied Health Sciences, School of Health Sciences, University of Birmingham, Edgbaston, Birmingham B15 2TT, United Kingdom; b Department of Nursing and Midwifery, School of Health Sciences, University of Birmingham, Edgbaston, Birmingham B15 2TT, United Kingdom; c Northumbria Law School, Northumbria University, Newcastle-upon-Tyne NE1 8ST, United Kingdom; d Chanon Consulting, Epsom, Surrey KT19 9TS, United Kingdom

**Keywords:** Sex worker, Healthcare access, Qualitative research, Thematic analysis, Health service provision, Enabling factors

## Abstract

**Background:**

Sex workers are frequently regarded by health services as a homogenous group whose health needs are related exclusively to the services they provide. Little research has explored sex workers’ health needs from their own perspectives.

**Aim:**

To understand their healthcare needs from their experiences and perspectives.

**Design:**

We undertook a four phase multi-method study to assess the health needs of sex workers in Birmingham, UK. This article reports the findings from qualitative interviews with sex workers (phase three).

**Settings:**

This study was conducted in the UK with sex workers living and/or working in Birmingham, UK.

**Participants:**

20 sex workers (10 men, 6 women, 2 trans/non-binary, and 2 not disclosed), were interviewed who were over 18 (21-40yrs): offering paid for sexual services involving in-person interactions. Online only service providers were excluded. Participants were recruited via partner organisations, personal networks, and social media (X).

**Methods:**

We undertook semi-structured interviews which explored healthcare needs and access for sex workers. Interviews were audio-recorded. Audio files were transcribed, and clean transcripts imported into NVivo to support data analysis. Transcripts were thematically analysed and mapped deductively against the adapted Andersen Model of Healthcare Service Utilisation.

**Results:**

Themes interpreted from interview analysis related to healthcare need, enabling factors, service provision and barriers to access. A further category “In an Ideal World” was interpreted inductively reflecting gaps in current healthcare service provision and access. Themes included: fear of judgement and stigma; social taboos; feeling unheard and dismissed by services (missed opportunities for engagement); judgmental service provision; and availability of tailored services and appropriately trained staff.

**Conclusions:**

Many sex workers experiences were underpinned by frequent exposure to psychological and physical trauma and violence, either related to lifetime experiences, experiences as a sex worker, and through contact with healthcare or support services. The intersecting marginalised identities of many sex workers were also a feature of their trauma exposure and interactions with healthcare staff. Recommendations include creation of holistic services; training of non-judgemental staff with insight into the complexities of sex work and the intersections of the multiple marginalisations sex workers face; appropriate training for staff in statutory services, e.g., General Practitioner (GP) primary healthcare practices; trained points of contact who can advocate and educate within their services; availability of peer support; integrated service pathways incorporating ‘fast track’ referral systems between partner organisations.

## Highlights

• Sex workers described multiple barriers to accessing healthcare services.
• Barriers included fear of judgement and stigma and social taboos around sex work.
• Barriers resulted in missed opportunities for engaging with healthcare services.
• Sex workers often experience trauma related to intersecting marginalised identities.
• Health services should be holistic and have insight into complexities of sex work.

**Contribution of the paper**

What is already known about the topic

• Sex workers are a frequently marginalised group who have specific healthcare needs
• Services which offer healthcare to sex workers predominantly focus on the ‘work related’ impact on health
• Little research has explored the health needs of sex workers from their own perspectives

What this paper adds

• Sex workers are a diverse group who have varied physical and mental health needs
• Sex workers often experience violence and trauma as a feature of both their working lives and their intersecting marginalised identities
• Sex workers face frequent judgment and stigma from healthcare services when trying to access healthcare for a variety of health needs
• The judgment and stigma which sex workers experience dissuades them from engaging with healthcare, and services should take a holistic trauma informed non-judgemental approach to counter this

Alt-text: Unlabelled box

## 1 Background

At the point this study was undertaken, limited research had focused on the wider health needs of sex workers (Martín-Romo et al., 2023). Much of the health research in this area has focused on; sexual health and sexually transmitted infection (STI); and the risks associated with sex work, e.g. drug misuse. including the conflation of – (particularly street) sex work with injecting drug use (Armstrong, 2018; Krüsi et al., 2016). Sex workers’ health is also put at increased risk due to experiences of violence, discrimination, and criminalisation (Deering et al., 2014; Harcourt et al., 2010; Krüsi et al., 2016; Platt et al., 2018; Rekart, 2005). This was highlighted during the COVID-19 pandemic particularly where a focus on sex workers and their health became highlighted, in the context of the pandemic and wider health issues of marginalised populations who continued to be more vulnerable (Callander et al., 2021).

Little research to date has explored fully how either the variety of services which sex workers provide impacts on their wider health needs, exclusive of, or additional to sexual health (Martín-Romo et al., 2023). Furthermore, there has traditionally been less focus on the needs of sex workers around wider healthcare and access to healthcare from their own perspectives, previous reviews have often for example, focused on delivery of interventions to sex workers (Johnson et al., 2023). This research focused on privileging the voices of sex workers in data analysis and interpretation, over the voices of academic researchers or health services providers and/or commissioners. Furthermore, society generally brackets sex workers into two groups; as either trafficked or otherwise exploited individuals with no agency, or as high-class workers who pick and choose their clients and live a life of relative luxury.

## 2 Introduction

Sex workers are frequently regarded by health services as a homogenous group whose predominant health needs are exclusively related to their job role and the services, they provide (Harcourt and Donovan, 2005; Ma et al., 2017; Martín-Romo et al., 2023). Sex work is defined in multiple ways as the provision of sexual acts, including full penetrative sex, in exchange for money or other goods or services (Campbell and Meth, 2024). Sexual services provided by a sex worker may include stripping, lap dancing, video pornography, telephone, or online services and physical contact with clients. There are multiple nuances in the way in which the wording around prostitution and sex work are used, and weaponised against sex work and sex workers (STELLA, 2013).

Sex workers health profiles are negatively impacted by not only their work, but in their position as one of the more marginalised and discriminated groups in society (Campbell and Meth, 2024). There have been historic gaps in research both with sex workers as a group, where they, similarly to other marginalised populations, have been considered ‘hard to reach’, when in fact they are a group who underserved and excluded from research, and health service design (Benoit et al., 2005; Elliott et al., 2002; Flanagan and Hancock, 2010; Fry et al., 2023; Garland et al., 2006).

The stigma and criminalisation associated with sex work industry, and specifically that directed at sex workers contributes to heightened risks of STIs and exacerbates sex workers’ exposure to and risk of violence and the need to conceal their professional identity (Armstrong, 2018; Platt et al., 2018; Potter et al., 2022). Violence against sex workers is endemic and may be physical and psychological and inflicted by clients, members of the public, protestors, and those in positions of authority, including police officers (Armstrong, 2018). The power dynamics between sex workers and their clients (Deering et al., 2014) combined with social discrimination and criminalisation (Hardy et al., 2016; Sanders, 2004) may further contribute to sex workers’ need for concealment, which in turn may impede access to appropriate timely healthcare.

This study was a commissioned project through Birmingham City Council focusing on assessing the healthcare needs of local sex workers. Local sex workers were defined as those living and/or working in Birmingham and providing sexual services to clients which involve physical contact. The study reported in this article was a work package as part of a wider study, which was a qualitative exploration of sex workers experiences of accessing healthcare services, using semi-structured interviews.

This is one of the few in-depth studies which explores sex workers’ health needs in relation to local access, commissioned by providers, from the perspective of sex workers. The use of community-based peer researchers is not a new phenomenon, but there has been limited work in this regard done with sex workers with equal consideration. Similarly to many other underserved populations, in part related to the over-research focus from the pandemic, there has been an historic and continued mistrust in research institutions (Elliott et al., 2002; Fry et al., 2023). This study was supported by two co-researchers with lived experience of being sex workers. These co-researchers contributed to many aspects of the study, but particularly the qualitative and ethnographic phases. Specifically, they were instrumental in accessing participants from sex worker communities and contributing to the analysis of qualitative data, and ensuring the framing and focus of themes was embedded in the nuanced lived experiences of those undertaking sex work, rather than from a service provision or academic perspective.

**Fig. 1:**
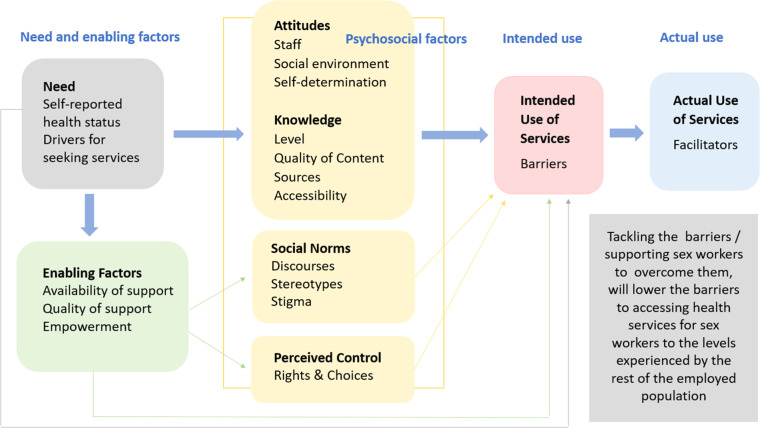
The Andersen model of access and utilisation (Adapted from Andersen, 1995).

The study was theoretically underpinned using the adapted Andersen Model (Fig. 1).

Theoretically, the study was underpinned by the Andersen Model of Access and Utilisation. The Andersen Model was employed in the SWAN project to help understand the complex factors influencing sex workers’ access to healthcare, particularly sexual health services. This model provides a structured framework for analysing why individuals do or do not use health services, taking into account both individual and systemic factors—making it especially relevant for populations facing multiple, overlapping barriers such as sex workers. The Andersen Model contains four psychosocial components: attitudes; knowledge; social norms; and perceived control. Anticipating that such factors would be significant for sex workers, we used a revised version (as presented) for the study.

These factors allowed the SWAN project to explore not only practical barriers like transport or clinic hours but also more nuanced challenges such as stigma, trust in healthcare providers, and discrimination. In this way, the Andersen model provided a robust theoretical foundation for a systematic, equity-driven exploration of healthcare access among sex workers.

## 3 Methods

### 3.1 Study aim

The aim of the qualitative work package as part of the wider multi-methods study was to explore and understand how sex workers experienced access to healthcare services.

### 3.2 Study design

This study was designed as a qualitative study incorporating one-to-one interviews with sex workers and focus groups with staff in services providing support and healthcare to sex workers. The focus of this paper is to describe how sex workers themselves describe their experiences and interactions of engagement with services, so the staff focus group data has not been incorporated into this reporting. Information around staff experiences is available in the published report (Bradbury-Jones et al., 2024).

### 3.3 Study setting

This study was conducted with sex workers living and/or working in Birmingham, UK.

### 3.4 Data collection and participant identification

Semi-structured qualitative interviews were undertaken with participants following a flexible guide. Semi-structured interviews are a frequently used method of data collection which allow for participants to relate their experiences around the phenomenon of interest, within a framework of the research focus (Dejonckheere and Vaughn, 2019; Jamshed, 2014). The guide (Appendix 1) focused on experiences of being a sex worker in the local area, the type of health care they had needed or wanted to access; if and how they accessed these health services; barriers and facilitators of health service access; and improvements to health service provision or design.

Participants were identified through purposive and snowball sampling. This happened in one of two ways. The first was that through partner organisations, potential participants could be identified and signposted to the research team or contact with the research team would be facilitated by the partner organisation. This signposting meant that any eligible sex workers identified by the partner organisations were given information about the study, via an introductory letter and information sheet, and contact details for the research team to be able to get in touch about participation, alternatively if a potential participant showed interest after receiving information from the partner organisations, they could direct the organisation to pass on their contact details for the research team to get in touch. This was dependent on personal preference. Second, potential participants could contact the research team after seeing the study advertised on social media, i.e., X (formerly known as Twitter).

### 3.5 Inclusion criteria

Participants who identified themselves as sex workers were eligible to participate in interviews if they were adults over 18 years old. Eligibility criteria around sex work were defined as providing sexual services/participating in sexual acts which involved physical contact with clients, as opposed to exclusively online or telephone work. Following consultation with local partner organisations, interviews were offered in English, or with interpretation support in Romanian, Portuguese or Spanish^1^. All participants requested to be interviewed in English.

### 3.6 Recruitment strategy

Initial purposive recruitment took place through the partner organisations identified prior to the study commencing. Partner organisations were asked to identify and approach service users and offer them information about study participation. Potential participants could then agree for contact details to be shared with the study team, or they could contact the team themselves.

Subsequent recruitment opened through multiple social media channels. This included sharing on X using sex worker networks and via mailing lists and messaging boards curated by varied online sources of support and information for sex workers. All participants were offered a £40 shopping voucher as a thank you gesture for their time commitment for participating in an interview.

Interviews were completed with 20 sex workers, all of which took place online via Zoom^TM^.

### 3.7 Ethical issues

Ethical review for this research was approved by University of Birmingham Research Ethics Panel (ERN_22-1376)19^th^ April 2023.

Given the sensitive nature of the research question, particularly around grey areas of legality of sex work in the UK, confidentiality and anonymity of participants was key to providing a safe space for participants to share their stories and experiences. Participants were given the option to use a pseudonym of their own choosing instead of their real name. They were also asked to create an ID code of their own choosing which could be used to trace their data if they wished to withdraw after participation. No participants chose to withdraw.

### 3.8 Data analysis

**Fig. 2:**
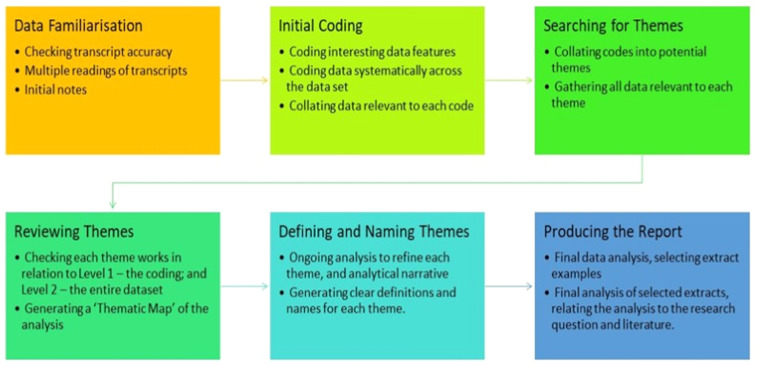
Codebook thematic analysis process according to Braun and Clarke (2016).

Transcripts were created from audio files of interviews with participants using a specialist transcription company who are compliant with UK Data protection legislation and requirements (Data Protection Act, 2018). Once cleaned and checked for accuracy, transcripts were uploaded to NVivo12^TM^ to support data analysis. The early transcript dataset was analysed using a mixed inductive/deductive approach, using the principles of Braun and Clarke’s approach to thematic analysis (Braun and Clarke, 2006). This approach is described in Fig. 2. These earlier transcripts which were created at the beginning of the data collection process were used to create the initial codebook, and later analysis was performed using this codebook and going back to these early transcripts to identify additional codes in the data.

This codebook was based on the Andersen Model of Healthcare Access and Utilization (Andersen, 1995) and was used to frame the analysis of the interview transcript data. Further inductive data analysis was undertaken alongside the codebook to capture further data and create themes which were both relevant to the overall study aims and highlighted as being important from the perspective of the co-researchers working on the study. The coding framework was discussed in conjunction with the wider project team, to ensure rigor and that the coding and thematic framework and more specifically with the co-researchers as codes and themes were developed from the data were reflective of wider experiences of sex workers and were framed from a sex worker perspective rather than from assumptions of the academic and/or health service provider perspective.

Within the write up for this manuscript, intended use of services is not included in the write up for this element of the overall study as that focused on services provided from a commissioning and provider perspective, not on how sex workers perceived and experienced their access and interaction with services.

## 4 Findings

### 4.1 Participant characteristics

The demographics of the 20 interview participants are reported in Table 1.

**Table 1:** Background characteristics of sex worker participants.

**Characteristic**	**Participants (n)**
**Age**	
18-20	0
21-25	5 (25%)
26-30	13 (65%)
31-35	1 (5%)
36-40	1 (5%)
40+	0
**Ethnicity**	
Asian – Pakistani	1 (5%)
Black – African	3^2^ (7%)
Black – Caribbean	(see^3^)
Any other Black Background (not specified)	4 (20%)
Any other mixed White Background	1 (5%)
Mixed background – Black African and White	7 (35%)
Mixed background – Black Caribbean and White	3 (7%)
White British (English/ Northern Irish/ Scottish/ Welsh)	1 (5%)
**Gender**	
Woman	6 (30%)
Man	10 (50%)
Trans*^4^(man or woman)	1 (5%)
Non-binary	1 (5%)
Preferred not to answer	2 (10%)
**Sexuality**	
Bisexual men	6 (30%)
Bisexual women	3 (15%)
Gay men	4 (20%)
Lesbian women	2 (10%)
Straight men	1 (5%)
Straight women	2 (10%)
Queer cis-man	1 (5%)
Queer non-binary person (assigned male at birth)	1 (5%)
**Language**	
English as first language	20 (100%)
**Relationship status**	
In a relationship	7 (35%)
Not in a relationship	2 (10%)
Preferred not to answer	11 (55%)
**Highest level of education achieved**	
O Levels/ GCSE/ CSE/ Scottish Highers/ Foundation Diploma	4 (20%)
AS or A Levels/ Advanced GNVQ	5 (25%)
Apprenticeship	1 (5%)
Degree (e.g., BA/ BSc)	7 (35%)
Higher degree (e.g., MSc / PhD)	1 (5%)
No formal qualifications	1 (5%)
Preferred not to answer	1 (5%)

2 One participant identified as Black African but also described themselves as Mixed – Black African and White and has been grouped with the mixed background for the purposes of population description.

3 Two participants who described themselves as Black Caribbean also identified as mixed White and Black Caribbean and have been counted in this grouping for the purposes of population description).

4 participants were asked about their gender, and asked if they identified as trans, i.e., gender different to sex assigned at birth, so the trans participants are also counted within their gender group.

**Fig. 3:**
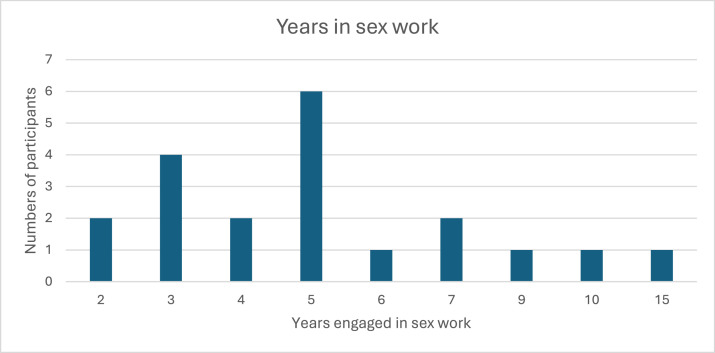
How long participants had been working as sex workers.

Interview participants had been engaged in sex work for an average of five years (mean 5.4; min 2yrs – max 15 yrs) (see Fig. 3). Most sex workers had been working for between 3 and 5 yrs (n=4, n=6, respectively).

Sex workers met clients (or had clients who found them) through multiple means, including online (n=19), at hotels (n=11), in massage parlours (n=8) and on the streets (n=3). They worked at different locations, including their own or clients’ homes, rented apartments or hotel rooms, private parties and in people’s cars (this was specific to street sex work).

### 4.2 Thematic findings

The themes interpreted from the interview transcripts were mapped to the Andersen Model of Access and Utilization. The model was used to frame the themes interpreted within the dataset as those related to:

An additional category was created named “*In an Ideal World”* this category included the suggestions from sex workers, about how services could be created and adapted to be more inclusive and accessible. These *ideal world* themes form the basis of many of the recommendations identified from this study. The psychosocial factors present in the model and as a framework for the themes created are those factors which are personal and psychological to participant as well as being socially constructed expectations and/or patterns of behaviour which relate to how a person interacts with the structures and within social expectations around them, In the case if this study this related to how sex workers felt they were perceived by society, and how this influenced individuals behaviours in relation to health and support seeking for themselves.

We would like to highlight that most of the sex workers in this study identified that they were sex workers through choice, rather than also being victims of trafficking (though within country trafficking was disclosed by one sex worker, who was able to move away from her trafficker, and continued in sex work of her own volition). When we describe ‘forced’ unsafe practices, these are in the context of experiences of sex workers who are talking about specific practices (see 4.2.1), rather than being forced into sex work by another person or persons.

#### 4.2.1 Psychosocial factors of needs

Sex workers reported that they, or their peers, were often forced into undertaking what they experienced as ***‘forced’ unsafe practices***. These ***‘forced’ unsafe*** practices were described based on client preference and were balanced against the financial need of the sex worker *versus* their perceived risk to safety. Other safety concerns were frequently health related, e.g., being in a financial position in which it was harder saying no to clients who were refusing to use barrier protection methods (condoms); or related to drug use with clients as part of the contact service (chemsex^2^). Sex workers frequently identified that their health-related needs encompassed that of ***mental health support*** which was challenging to access. These needs were multi-faceted and varied for different sex workers. Some felt that they needed mental health support to manage their struggles with self-esteem in relation to the occupation.

“It’s never really easy at the beginning. There are a lot of emotional traumas, a lot of doubts within yourself if you’re doing the right thing.” S05

Others wanted support for sexual assaults and abuses which they had experienced during their sex work but felt that because of the occupation, these assaults and abuses were not taken seriously. Similarly, other sex workers disclosed underlying mental health issues in relation to adverse childhood or adolescent experiences, these ranged from sexual abuse, parental death, or family alienation related to the sex workers’ sexual orientation. These intersectional characteristics of many participants was felt to be misunderstood by practitioners in physical and mental health support. Sex workers felt they were seen as being on the lowest rung of the societal ladder, where the influences of individuals’ multiple marginalised identities, including ethnicity and sexual orientation intersect and compound each other.

“There needs to be mental health services, like counselling as well as proper therapy for things in our past as well as now. They need to assess our emotional state to help with the impact of rape and of being a sex worker.” S21

Sex work has a wider impact on health that is not just related to sexual and mental health, although sometimes these were linked. Being a sex worker was perceived to be a risky profession related to accessibility for wider support, like permanent safe housing, and the impact of homelessness then further exacerbated both the risk of sex work and the need to continue in sex work as a quick means of financial support. Participants identified the lack of permanent safe housing as a structural barrier to accessing healthcare (see 4.2.3), as well as the risks inherent in the profession of being a sex worker (see 4.2.3 – ***appropriate service provision***).

#### 4.2.2 Psychosocial factors which mediate access

The psychosocial factors identified which mediated access to healthcare included both internalised and enacted stigma, attesting to the complex psychosocial world that sex workers must navigate in their daily work and personal lives. Where sex workers accessed ‘standard’ healthcare services they were unlikely to disclose their occupation to the people treating them. This was rooted in a deep-seated mistrust of healthcare services being able and willing to treat them without judgement. Sex workers all described the ***fear of judgement and stigma*** which led to them accessing healthcare in a piecemeal way – moving between services and locations; deliberately withholding information about their experiences; or avoiding seeking healthcare altogether.

“It took a lot of procrastination and having to encourage myself, and some depressive phases to be able to finally go and seek out mental health support, and even at that it was the first three sessions was difficult, because I found it hard to say really what I was doing. I was explaining to the therapist this was a problem I have, but I wasn’t willing to tell him… tell them it was because of starting sex work.” S05

“I’d been sexually assaulted by a client. I know something was wrong down there, but because of the whole circumstances, and because I’d been working, I said I can’t go to the police, can’t go to the doctors, because they’re all going to look at me and say it’s my fault.” S11

The stigma and prejudice that sex workers face was also perceived to be based on and reinforced by ***social assumptions about sex workers*** which they identified as how society views them. This related to sex workers feeling like the unseen and unwanted members of society – or not as part of the community at all.

“They look down on people, the fact you do sex work, and it’s the same thing with people who want to change genders, because I’ve had people say stuff about sex workers, how dirty they are and how they spread diseases like that, but which isn’t the case” S01

“…the stigma attached to the profession. Most people look at sex work as some form of depraved occupation where the lowest of lows engaging just to get by” S12

Although sex work as an occupation is seen as a taboo topic, many of the participants in this study also faced intersectional prejudices, particularly in relation to their sexuality, which exacerbated their isolation from society and fear of support seeking from mainstream healthcare services.

“I will not be open about that, because of a lot of people have a lot of bias. A lot of people are not very accepting of sexuality that is different from the mainstream. A lot of people look at us as people with mental issues, and it’s fully biased, it’s difficult when you have to face some bias and treatment because of your sexual orientation. So I just try to keep a bare minimum, I just tick the straight option.” S06

“The sexual health clinic staff need to be more open-minded about Black people and about sexual orientation. I don’t think they are comfortable about me as a Black person or a Black queer sex worker. I think they are judgmental about Blacks, and also that they have negative views about Black men” S21

#### 4.2.3 Barriers to healthcare access

Sex workers felt their ***ability to seek support*** was impeded by multiple factors, e.g., over-subscribed services, or the actual set up of service delivery, e.g., specific locations for collection of certain prescriptions. This was specific to sex workers on methadone for drug use, where prescriptions would only be dispensed in the area where drug dealing was prevalent. This had the ability to make services easier to access for those who remain in the area but created additional barriers and challenges for those who have moved out of the area to try and escape that element of their life. These elements combined with other barriers, such as those created by a lack of permanent housing, or having to move frequently between areas, impacting people’s ability to register with GP surgeries.

“I don’t go to the GP. I only ever have temporary housing, so I wouldn’t be able to register at a GP; but I wouldn’t go anyway. I would never tell a GP or another health person that I was a sex worker. I haven’t been to any other health services.” S21

For many sex workers the emotional labour involved when seeing different staff members and having to continually retell their story or disclose their sexuality and/or occupation, also acted as a considerable barrier to accessing healthcare.

I was on mental health medication they were forcing me to go into the surgery every two weeks to make sure I’m okay on my medication, and it was like it was different doctors as well, because it’s not a set doctor there, it’s like stand in. What do they call them?

Interviewer: Like a locum GP?

“Yeah, that’s the one, no it’s the locum that comes in. So, it’s like you’re telling one doctor one thing, and then you’ve got to start all over again to another doctor. We don’t need that, it’s hard for us to be explaining our story… having to keep going over it with different people, because they can’t be bothered to read the files.” S11

Even when accessing healthcare, sex workers feel they are met with ***judgmental service provision*** from their healthcare providers, in which the social perceptions of sex work and sex workers are reflected in how they are treated by staff within the healthcare setting. This links to the psychosocial factors of ***fear of judgement and stigma*** (see 4.2.2), where sex workers in this study described the psychological fears of being treated as other, which created barriers to engagement with healthcare, but is based in experiences of treatment by healthcare staff where provision was perceived as judgemental, thus creating additional barriers where their fears are proved an experienced as reality. This also applied in settings which were ostensibly for the most vulnerable members of society.

“You’ve got the [name] Centre doctors, which is meant to be for vulnerable adults. Well, some of the staff in there, like reception staff, speak to you like shit, treat you like shit. They look you up and down like you’re nothing.” S11

Many sex workers highlighted that services which are available are often not ***appropriate services.*** This may be related to the type of support on offer, the wider client/service user group the service is intended for, for example victims of sexual violence; or the ***physical access, e.g., time and location*** which does not account for the variety of sex workers and their locations.

“Where it is and also the times, because they’d have an early morning slot, and the thing is I find it really hard to travel all the way to the centre, and then getting up, because you’re working all night, and getting up in the morning first thing, and then going there, and then that was a struggle.” S01

“The thing about mental health support is a lot of them are just really bad, they’re just they’re either just went to school, just read some books, and then not… yeah or they’re just not… I don’t know. It’s very specific types of any sort of help that you need. Many people are like do trauma work but then also do… so have some idea of what you’re doing. Because I’ve been to people, I’ve had therapy with people that don’t have, and they’re like oh I can do it, so you go there and you’re like you don’t really know what it’s like” S02

#### 4.2.4 Actual use of services

Sex workers were most frequently using the tailored sexual health services which were either open to all or specifically designed for sex workers. They described frequent positive interactions with the services designed fo either sex workers, or marginalised groups, e.g., LGBTQ+ groups, because of the willingness of staff to go ***above and beyond service provision***.

“I sort my things out through the ISVA [Independent Sexual Violence Adviser], and sometimes the nurse at the sexual health clinic she helps me out even with my housing and everything like that. She’s a sexual health worker, but then she goes out and beyond as well.” S01

However, in their use of other services, there was a conscious decision made to ***avoid information sharing***, particularly in relation to the specific circumstances of why they may be engaging with a service.

“I tend to keep it to a minimum, maybe a service on the last part I have no option than to disclose it then I disclose.” S15

Sex workers who accessed healthcare often left the service feeling ***unheard and dismissed*** whether they had disclosed about their sex work, or other forms of abuse or not. This dismissal often resulted in feeling that the services and staff had ***missed opportunities for engagement*** with the sex workers, which could have led to an increased likelihood of further engagement in the future.

“He [abusive partner/trafficker] told them, he was like, ‘When I get home I’m going to kill her’ and they heard that and they didn’t do anything. […] That’s kind of like the one incident that stands out to me in terms of healthcare and not getting enough support. They could see what he was like, and they didn’t do anything. They did question me, they were like… “Oh is everything okay and stuff?” And obviously I’m not going to say anything, because I’m just not” S02

Many sex workers described developing a ***personal focus on health*** which in turn had an impact on their working practices. This focus of sex workers on their health, conflicts with the stereotypical social narratives around sex workers sexual health status, as a reservoir for sexually transmitted infections, and overall lack of self-care, as part of the narratives around sex work as a means to an [drug addiction] end. The sex workers in our study described how prioritising their health was key for both their own health, the health of their clients, and to maximise their working time.

“At the moment I take my health more seriously. I don’t do some of the things I did when I was starting out. I get myself tested about once a month, just to keep my body in the right shape, engaging in exercise I also try to eat healthy. So because this occupation is sex work, it doesn’t mean I would look so low of myself.” S12

“I actually care very much about my health. So I make it… I would just need to get myself tested monthly, like a full body check-up. Also during the acts I make sure to use protection as much as I can, because as I care for my health I also care for the health of my clients, and also my potential clients. So I usually wouldn’t let desires of one client jeopardise my health status, and status of my prospective clients. So, I usually don’t do anything without - always with protection, and also I typically go to check-ups.” S05

A smaller number of sex workers reported that access to some healthcare services, was available at the same level of access as the rest of the population. This indicates that for some they feel their access to healthcare is neither better nor worse than the general population. Though of the 20 interviews, only two identified this, and for one [S01] this was specifically related to dentistry.

“I feel I got the level of access every other person had” S06

#### 4.2.5 ‘In an ideal world’

Sex workers were asked about how to improve services, and what was missing from the services they currently access. The responses were interpreted and created an overarching theme which is described here as ***in an ideal world.***

“If I have the opportunity to design a service on healthcare, I need I think the judgement part I think I would really, really love to take, because people out there are judging, and judging, and judging, and they don’t know what it’s like to be in your own shoes and that. So design a healthcare that I think won’t really matter what you do, and just your service is first, your health first.” S15

A need within services which was highlighted by all interview participants was a lack of ***appropriately trained staff*** who could offer empathetic, non-judgemental healthcare.

“You don’t need to fully understand, you don’t need to have lived experience of it. But you need to have a lot of experience of working with women that have been through that, and read a lot about it, and just being informed about it. I think there’s a lot of support missing in that aspect.” S02

“The proportion of health workers who actually know, who are being employed, that is do know about such jobs, and not as much as other health workers. So I can actually find it I actually get someone, a health worker who is actually going to understand your problem. So I think people like us or people who have experience like us, health workers who have experience like us should be included, should be employed into this system, so services can be much more easier.” S08

Many sex workers also identified that to increase service access, ***increasing visibility and availability*** of services would support their access. This was related to visible advertising of sex worker specific services. This would act in multiple ways – advertising the service, raising the visibility of sex workers as a valid population group with their own needs, and flagging the location of the advert (e.g., inside a GP surgery) as a ‘safe’ place for sex workers. The other aspect of service access related to widening access, and providing varied options for accessing services, including service capacity, peer led groups, and online booking, or online options for accessing support groups.

“When I say there’s room for improvement, in a sense that things could be done even more digitally. Yeah, things could be done even more digitally, like setting up… Just go online, go to the site of the clinic, and book an appointment, and through that there wouldn’t be stress of going through calls or going to the clinic first before you book an appointment, then you have to come back” S08

Although the focus of this research was about access to healthcare, many participants identified gaps in the ***legal and justice support*** which they felt was available to them. Sex workers frequently feel that because of their occupation they are treated as and seen as less valuable than other members of society. This impacts healthcare access but also reflects the lack of legal protection and justice that they feel they have access to, as the lack of legal oversight, leaves sex workers at higher risk of exploitation.

“We’re human beings and these activities we’re not really hurting anyone. It’s an activity that it’s consensual between consenting adults. So, I feel if government legalises, and I wouldn’t say decriminalise, on the more aspect of it not to look and see it as something very bad or something like that. Because I feel once it’s legitimised it’s going to provide better protection and security for sex workers, and it’s also going to provide inclusiveness for everyone.” S06

Participants all highlighted how important the role of ***peer support*** is and can be for both supporting mental health and being able to talk to people who have in-depth understanding of the nuanced reality of sex work.

“People I know do the same job we tend to hang out some […] and try to support ourselves, and share some experiences like I am doing now, and it’s quite helpful being people like I said where you can be free, there is no judgement there and all that, it’s very helpful.” S16

This was part of the call for specifically ***tailored services*** which should be run, not only by staff with appropriate knowledge, training and empathy.

“I would like to visit the healthcare service which I know the practitioners they are really, really, really understanding and open-minded to me, and can relate on a very deep level with me” S16

This should also include services which are designed to offer safe spaces and consider the various nuances of different types of sex work and that there is not likely to be a ‘typical’ sex worker, or that sex workers have the same needs as the rest of the population, even when requiring similar support, e.g., after having been subjected to sexual assault.

## 5 Discussion

The positive findings from this study show that the health services available to sex workers in Birmingham are well regarded by many who use them. We heard reports of staff within these services, frequently going ‘above and beyond’ to meet the needs of sex workers. A particular feature was signposting into other services. Services which specifically cater to marginalised populations, including the LGBTQ+ population are also well regarded, and their low threshold for access combined with the less formal clinical offering for testing and access to prophylactics, makes them a less threatening prospect because of the minimal amount of personal information which is documented, recorded, and shared across the health service.

The positive narrative is heavily outweighed however, by the pervasive stigma and judgement of how sex work is perceived from both society and from staff who offer general healthcare services. There was an understanding among many staff participants that sex workers need access to the mental health support, which is provided by some of the services, but that the need outstrips the current levels of service provision and that wider mental health services do not understand the nuances of the sex worker population to offer appropriate support, or the risks which they feel forced into taking. For example, sex workers described perceptions of having to participate in riskier sexual behaviours, e.g., unprotected sex at client request due to financial insecurity, where not engaging results in lost income, or where engaging results in substantially increased payments (Shannon and Csete, 2010). The sex workers echoed this finding themselves. Sex workers described being unable to disclose their profession which created barriers to the therapeutic relationship with their counsellors, and described how accessing standard services for sexual violence and assault still left them marginalised and unsupported where there were perceptions of blame or expectation of violence due to the nature of sex work (Argento et al., 2021; Singer et al., 2021; Strega et al., 2014). The rest of the discussion also highlights the recommendations for service design.

Sex workers described a need for holistic services that encompass statutory health care alongside mental health support delivered by trained lay or peer staff who are non-judgemental and have insight into the complexities surrounding sex work (Campbell, 2000; Potter et al., 2022). They identified that staff training should focus on intersectionality’s of marginalisation and discrimination which sex workers face both due to their occupation and the social assumptions about sex work, including the gendered assumptions about those who undertake sex work (Matthen et al., 2018) but also due to their other personal characteristics, e.g., ethnic background and/or sexuality or gender. The intersectionality of sex workers identities and how these interact and further marginalise individuals has been well researched in recent years (Hunt et al., 2017), and has implications for service design (see Table 3).

Participants articulated that their mental health support needs were related, in part, but not exclusively to their lived experience as sex workers as reported in other studies (Brookfield et al., 2020; Puri et al., 2017; Reynish et al., 2021). Mental health support for sex workers was described as needing to be offered as specific to sex workers daily lived experiences of violence and judgment. Furthermore, sex workers in this study called for specific mental health support for the impact of rape, sexual assault and abuse as a sex worker, where they felt that general support for survivors of sexual violence was dismissive of sex workers need for support (Martín-Romo et al., 2023). For some sex workers, historic abuse and trauma had influenced their route into sex work, but this past trauma was outside of the remit of the services which do provide mental health support to sex workers, as this support was tailored and specific to current trauma related to sex work (Table 2).

**Table 2:** Themes identified which align with the areas identified in the adapted Andersen Model of Healthcare Access and Utilization.

*Category from Andersen Model*	*Sub theme*	*Sub theme description*
***Psychosocial factors of needs***: underlying needs which influence help-seeking which are inherent to sex work	***Forced unsafe practices***	Often, but not exclusively, sexual practices which some sex workers feel compelled to accept where they are in a more precarious financial position, e.g., lack of condom use for higher fee, even where this is not something they want to agree to.
	***Mental health support***	The impact on a person’s mental health of engaging in sex work, which is a role in which violence is endemic, and the mental health needs which may be underlying a person’s life experiences and journey to sex work, for example, being ostracised by family and community for sexual orientation.
***Psychosocial factors which mediate access:*** influences on sex workers perceptions of how to or whether to access services for healthcare	***Fear of judgement and stigma***	Sex workers feared the reaction of those in health services when they disclosed their profession, and thus chose to not disclose as a safer option than risking being stigmatised and losing either access to services or the feeling of being cared for and seen as human. This was anticipatory fear of judgement.
	***Social assumptions about sex workers***	The way in which sex workers described feeling othered by society on a general scale, and that people who engage in sex work are considered unclean or depraved in some way.
***Barriers to healthcare access:*** things that made it harder for sex workers to access services	***Ability to seek support***	This describes how confident sex workers were in seeking support. This might be related to service structure and availability of appointments or may be about the emotional labour of reiterating their story to multiple HCPs.
	***Judgmental service provision***	This linked to anticipated fear of judgement and stigma but was related to the actual judgement experienced by sex workers within services.
	***Appropriate services***	The appropriateness of services was linked to whether services were deemed to be understanding of the needs of sex workers, for example some sex workers accessing sexual assault services felt they were still treated as less deserving of support because of the lack of understanding from other service users and staff about trauma experienced by sex workers.
	***Physical access to services***	This related to sex workers capacity to access services where the location was challenging, and the appointment system created barriers i.e., drop-in clinics with no pre bookable appointments which ate into working time.
***Actual use of services:*** how sex workers engaged with the services they used	***Above and beyond service provision*.**	This was described as the ways in which staff in services frequently appeared to work beyond their commissioned remit, to provide a holistic, compassionate service to sex workers.
	***Avoiding information sharing***	This described how sex workers when engaging with different services, e.g., emergency healthcare, would avoid disclosing their job, and sometimes this included avoiding disclosing how they had experienced violence or injury because of the fear o stigma and judgement
	***Feeling unheard and dismissed***	When some sex workers did disclose information related to trafficking, rape or abuse by partners or others in their lives, e.g., a pimp, this was frequently felt as being dismissed by the healthcare worker and led both to missing opportunities and to future avoidance of information sharing
	***Missed opportunities for engagement***	Similar to feeling unheard, this related to the near misses’ where sex workers could have disclosed and sought help but felt that they would be unheard so chose not to.
	***Personal focus on health***	How sex workers described their need and wish for health seeking support, where they were as, or more conscious about staying healthy generally, in contrast to older social tropes around sex workers and STIs, for example.
***In an ideal world***: sex workers perceptions on good service provision		Describes how services could be designed from a sex worker perspective to be more appropriate, inclusive and accessible for their community.

Moreover, the stigma and judgment which sex workers described facing and anticipating in their encounters with healthcare services is likely to compound their trauma (Jennings, 1997). These layers of stigma, prejudice and having to relive experiences when meeting new healthcare professionals may result in interactions with healthcare providers being traumatising in and of themselves (Potter et al., 2022). These nuances do not only relate to sex work itself and the highs and lows, but also the fact that many sex workers themselves have multiple marginalised characteristics such as sexuality, gender, and ethnicity, as well as their occupation(Puri et al., 2017; Reynish et al., 2021). These intersecting elements of sex workers’ identities combine to create additional barriers to overcome when disclosing and seeking support initially, and then in the process of potentially having to repeat their account, and thus relive their trauma, to multiple professionals, or to reception staff when booking appointments ((US), 2014). The related implications for practice are shown in Table 3.

**Table 3:** Service delivery recommendations and implications for practice.

1. Holistic services that encompass statutory health care alongside mental health support delivered by trained lay or peer staff who are non-judgemental and have insight into the complexities surrounding sex work. 2. Specialist services which are tailored for sex workers with different experiences, e.g., survivors of sexual violence. 3. Trained points of contact who act as advocates and educators within their services, for other wider services, e.g., housing, or police. 4. Training for staff in standard statutory services, e.g., GP, ‘normal’ GUM clinics, so they have a better understanding of sex work, and sex workers and may be able to provide care which is empathetic and lacking in judgment. 5. Integrated service pathways which can offer ‘fast track’ referral systems between partner organisations, more informed and empowered information sharing by sex workers, and also remove the need to disclose their occupation and needs every time they encounter a different service. 6. Multiple options for sex workers’ engagement with health and support services, including offering online support and booking options. 7. Opportunity for peer-to-peer engagement for support and sharing of common frameworks of understanding life as a sex worker. 8. Creating a ‘safe to disclose’ training scheme which is also advertised and ‘flags’ services as offering non-judgmental, trauma informed support and has specific routes for support for people who are sex workers

Sex worker participants described feeling at the edges of society, and that they were frequently ignored, marginalised and as though local communities would prefer, they were not part of them (Hunt et al., 2017). These perceptions may also be reflected in how services for sex workers are designed and promoted and have been shown to also influence the level of violence which sex workers might face (Krüsi et al., 2014). There appears to be limited or minimal public advertising of services. Word of mouth recommendations and warnings about services which offer good or poor care travelled fast. Many people use services with a good local reputation, which increases the pressure on those services, in terms of service capacity and subsequent access for service users. This also reflects the gaps where sex workers are uncertain about which services are ’safe’ to access until this information is shared by their peers (Singer et al., 2021). There are inherent risks in advertising ‘safe’ services for sex workers, could increase stigmatisation where sex workers accessing these services become more visible when seeking support. We would suggest that how these services are advertised, is designed in conjunction with sex workers would make them safe at the point of access. For example, visible signage in general areas, indicating staff have been appropriately trained, as opposed to designated clinics for sex workers which increase their public visibility.

Sex work can by its nature be an isolating profession; there are legal restrictions on sex work and shared housing/ working spaces, for example, where a house, or premises has sex workers providing paid for services, even where this ia a room let by a landlord (Brouwers, 2022; Griffiths, 2025), this constitutes the legal definition of a brothel, which are illegal in the UK. This results in sex workers who are isolated from each other, either for fears of their own legal safety, or by those running the premises, to reduce their own legal risk . Alongside social stigma, and judgement, meaning people are unsure who they can safely share information with (Benoit et al., 2020; Singer et al., 2021; Toubiana and Ruebottom, 2022). Peer support has been highlighted in this study and elsewhere as supportive of both mental health of sex workers and influencing access to healthcare services (Singer et al., 2021). Wider advertising of sex worker specific services was suggested as a way of highlighting and increasing the visibility of services that are available as well as acting as a flag or marker that the places advertising services for sex workers might be more likely to be ‘safe’ spaces, in the same way that rainbow lanyards and similar imagery identify LGBTQ+ supportive staff and locations. This would rely on the advertising location (e.g., GP surgery) having appropriately trained, non-judgemental staff. The red umbrella image is one which was highlighted by our co-researcher advisors as an image which indicated sex worker positivity and may be a consideration for this kind of ‘mark’. See Table 3 for related implications for practice.

### 5.1 Strengths and limitations

While we put in place the structures and ethical approvals to conduct the interviews in multiple languages, all sex worker participants had English as their first language. Lack of non-English speaking sex worker participants may be less representative of hidden populations of sex workers, particularly those who may have been trafficked within, or between countries. Similarly, a larger proportion of our participants identified as men. Though it does give specific insight into the experiences of men who have sex with men in the course of sex work, this may limit the generalisability of our findings to the wider sex worker population. However, we regard the recruitment of 20 sex workers to the study as highly successful, when matched against our anticipated numbers. This is largely attributable to recruiting through the ‘insider’ networks of one of the co-researchers and is a testimony to the strength of researching within a co-produced framework with experts by experience.

This qualitative study aimed to privilege the voices of the sex workers above that of either the staff providing or commissioning services, and the wider literature which looks at services and interventions which have been in use or commissioned for use, and by doing so focused on what the sex workers own perceived needs and assessment of their access to healthcare looked like. All this was with reference to the study’s theoretical framework, The Andersen Model. We found its domains to be appropriate and helpful in organising the volumes of rich qualitative data arising from this study.

One other limitation is the focus on in-person sexual service provision. This was a pragmatic decision made partly due to commissioning of the study with a focus on local services and also due to the restrictions we placed upon yourself in terms of participant confidentiality and anonymity we may have increased the risk of recruiting impostor participants. However, this does mean it is more challenging to understand whether similar barriers to accessing healthcare exist, for those who operate exclusively online.

This research was designed with a focus on embedded co-production with co-researchers with lived experience of sex work. Aside from impacting positively on recruitment, this strengthened both the design of the study and supported recruitment through varied networks which are specific to people working in that space. The co-researchers also supported with data analysis, and triangulation of findings in relation to analysis and suggestions and recommendations for practice. In particular, the focus on the *ideal world* was considered by the co-researchers to particularly be a novel approach to exploring and considering health service access and needs of sex workers, where much of the literature and research in this area has focused on barriers and facilitators to access in the context of legal redress and legitimisation of sex work.

## Implications for practice

From the findings, we have identified eight key areas that translate into service delivery recommendations and implications for practice (Table 3).

## CRediT authorship contribution statement

**E. Molloy:** Writing – review & editing, Writing – original draft, Validation, Project administration, Methodology, Investigation, Formal analysis, Conceptualization. **R. Tiwana:** Writing – review & editing, Project administration. **B.c French:** Writing – review & editing, Formal analysis. **C. Christie:** Writing – review & editing, Validation, Methodology, Investigation, Formal analysis. **H. Smailes:** Writing – review & editing, Project administration, Investigation. **J. Taylor:** Writing – review & editing, Validation, Supervision, Project administration, Methodology, Formal analysis, Conceptualization. **C. Bradbury-Jones:** Writing – review & editing, Visualization, Validation, Supervision, Project administration, Methodology, Investigation, Funding acquisition, Formal analysis, Conceptualization.

## Declaration of competing interest

The authors declare that they have no known competing financial interests or personal relationships that could have appeared to influence the work reported in this paper.

## Appendices

### Appendix 1. Interview guide for Sex Worker participants

Interview Guide (Semi-structured interviews with sex workers)

**Sex Workers Assessment of Health Needs in [CITY] (SWAN)**

**Introduction (after signed consent)**

My name is <<<INSERT NAME>>, and I am here to ask you about your experiences of sex work in [CITY], and your experience of accessing or needing to access any support and health services.

Recap of the rules of the interview: e.g., they can stop the interview at any point, remind participants that they are in control of what they say and remind participants that anything said will remain confidential unless there is a safeguarding risk.

Key topics to be covered

1. What is life like for you as a sex worker in [CITY]?
  a. *Prompts: did you grow up here? How did you come to be a sex worker?*
2. What kind of health or support services have you needed to access in the past?
  a. *i.e., GP, Screening services, dentistry, hospital outpatients, pregnancy, menopause, eye tests, hearing*
3. If yes to services being used, can you tell us more about the service and how you interacted with them?
4. What made it easy for you to access services and what made it difficult?
  a. *i.e., cultural influences – stigma around sex work*
5. What do you think is missing from the services which are currently available to you?
6. Anything else you would like to add about your experiences and thoughts?

Thanks, and close

## Footnotes

1 Initial criteria for inclusion specified all interviews would be held in English, but following consultation with the funder, and after seeking additional ethical approvals, the option of interviews in the three most commonly spoken languages supported by the telephone interpretation service was added (June 2023).

2 Chemsex also known as wired play or PnP (Party n Play) is the consumption of drugs to facilitate or enhance sexual activity
